# The associations of psoas and masseter muscles with sarcopenia and related adverse outcomes in older trauma patients: a retrospective study

**DOI:** 10.1007/s40520-022-02119-7

**Published:** 2022-03-31

**Authors:** Surabhi Varma, Michael S. J. Wilson, Mitesh Naik, Amandeep Sandhu, Helen Chidera Uchenna Ota, Christopher Aylwin, Michael Fertleman, George Peck

**Affiliations:** 1 West Middlesex University Hospital, Chelsea and Westminster NHS Foundation Trust, London, UK; 2 Forth Valley Royal Hospital, NHS Forth Valley, London, UK; 3 Imperial College Healthcare NHS Trust, London, UK; 4 Northwick Park Hospital, London Northwest University Healthcare NHS Trust, London, UK; 5 Department of Medicine for the Elderly, St Mary’s Hospital, Mary Stanford Wing Praed Street, London, W2 1NY UK

**Keywords:** Sarcopenia, Psoas, Masseter, Elderly trauma, Frailty

## Abstract

**Background:**

There is an emerging role for radiological evaluation of psoas muscle as a marker of sarcopenia in trauma patients. Older trauma patients are more likely to undergo cranial than abdomino-pelvic imaging. Identifying sarcopenia using masseter cross-sectional area (M-CSA) has shown correlation with mortality. We sought to determine the correlation between psoas: lumbar vertebral index (PLVI) and the M-CSA, and their association with health outcomes.

**Methods:**

Patients aged 65 or above, who presented as a trauma call over a 1-year period were included if they underwent cranial or abdominal CT imaging. Images were retrospectively analysed to obtain PLVI and mean M-CSA measurements. Electronic records were abstracted for outcomes. Logistic regression methods, log scale analyses, Cox regression model and Kaplan–Meier plots were used to determine association of sarcopenia with outcomes.

**Results:**

There were 155 eligible patients in the M-CSA group and 204 patients in the PLVI group. Sarcopenia was defined as the lowest quartile in each group. Pearson’s correlation indicated a weakly positive linear relationship (*r* = 0.35, *p* < 0.001) between these. There was no statistical association between M-CSA sarcopenia status and any measured outcomes. Those with PLVI sarcopenia were more likely to die in hospital (adjusted OR 3.38, 95% CI 1.47–9.73, *p* = 0.006) and at 2 years (adjusted HR 1.90, 95% CI 1.11–3.25, *p* = 0.02). Only 29% patients with PLVI sarcopenia were discharged home, compared with 58% without sarcopenia (*p* = 0.001).

**Conclusion:**

Sarcopenia, defined by PLVI, is predictive of increased in-patient and 2-year mortality. Our study did not support prognostic relevance of M-CSA.

## Background

Sarcopenia has emerged as a pivotal concept in research and clinical practice due to its correlation with frailty and its association with adverse health outcomes across a spectrum of patient populations. It is defined as “muscle failure” characterised by loss of muscle strength, quality and quantity [1]. A variety of physical performance, anthropometric and diagnostic imaging tools have been used to measure sarcopenia in research and clinical settings [2]. The role of computed tomography (CT) to assess muscle composition has gained popularity and is favoured for its routine diagnostic use in many specialties, including surgery, trauma and oncology, with availability greater than other modalities, such as magnetic resonance imaging (MRI) and dual energy X-ray absorptiometry (DEXA).

Assessment of sarcopenia using CT in older trauma populations has been associated with increased length of stay [3], in-patient complications [3], mortality at 6 months [4] and 1 year [5] and discharge disposition [6]*.* Most studies to date have used psoas cross-sectional area (P-CSA) to assess for sarcopenia [7]. This relies upon cross-sectional imaging of the abdomen and pelvis.

However, seventy-five percent of trauma in the elderly involves the head and neck. Many older trauma patients have isolated cranio-cervical injuries not always requiring cross-sectional imaging of the abdomen and pelvis. Identifying alternative targets for opportunistic sarcopenia measurement in older trauma is therefore warranted to enable its pragmatic application towards assisting decision-making, prognostication and discharge planning. The masseter muscle has been posited as a suitable alternative given it is sizeable, superficial and therefore, suitably quantifiable on routine CT neuro-imaging. It has been used to predict outcomes in carotid endarterectomies [8, 9] and strokes [10].

Specific to trauma, 4 studies have looked into masseter area measurements as a prognostic parameter [11–14]. In 2016, Wallace et al. introduced the role of the masseter as a sarcopenia surrogate in a large retrospective cohort study in trauma patients aged above 65 years [11]. They reported that masseter CSA (M-CSA) was a more robust independent indicator of cumulative 2-year mortality, after adjustment for other variables, compared to P-CSA. Another study demonstrated that masseter sarcopenia was associated with increased 1- year all-cause mortality in older trauma patients admitted to intensive care. [13]. However, this association was clinically significant only when there was co-existing brain atrophy measured using the bi-caudate ratio. Masseter sarcopenia in severe traumatic brain injury has also been shown to be predictive of 30-day mortality and the need for longer-term acute facilities or rehabilitation on discharge [12]. However, a recent Korean study involving patients with traumatic brain injury of all ages negated statistical prognostic value of masseter measurements, but supported the correlation of psoas measurements with mortality and Glasgow outcome score [14].

We hypothesise that masseter CSA is comparable to psoas CSA in predicting health outcomes in older trauma patients. Thus, the primary aim is to determine whether masseter and psoas sarcopenia are associated with mortality up to 2 years in patients aged 65 and older admitted with trauma. Our secondary aims evaluate the association of masseter and psoas sarcopenia with in-hospital outcomes including in-patient mortality and discharge destination. This has not been adequately evaluated in previous studies. While composite 2-year mortality is an important calibration, these intermediate outcomes may be more clinically relevant to patients and physicians. We have also attempted to quantify the strength of correlation between M-CSA and P-CSA.

## Methods

### Subjects and study design

This retrospective cohort study was approved by the Health Research Authority. St. Mary’s Hospital is the major trauma centre for Northwest London. Our local Trauma and Audit Research Network (TARN) database was used to identify consecutive patients aged 65 years or above who were admitted with suspected traumatic injury over a 1-year period from October 2015 to October 2016. Patients who did not sustain any injuries or whose scans were insufficient due to poor image quality or resolution were excluded. Patient demographics are routinely recorded in the TARN registry. A retrospective review of CT scans and analysis of electronic medical records for in-patient outcomes was performed. Mortality data were extracted from Summary Care Record via Spine portal, unless captured in the Trust’s electronic patient record system.

### Sarcopenia quantification

The dependent variables of interest were the M-CSA and the psoas:lumbar vertebral index (PLVI). Measurements were obtained by two radiologists, who were blinded to patient outcomes. Images were analysed using Carestream Picture Archive and Communications System software. Average M-CSA was the mean of measurements on each side deduced from the longitudinal axis 2 cm below the zygomatic arch. This requires reconstructing the imaging plane to align with the proximal and distal attachments. Patients who did not have a CT head or in whom bilateral measurements could not be computed were excluded. P-CSA was measured bilaterally at the level of L4, just below the origin of the posterior elements, and averaged. Height and weight are not always readily available in the hyper-acute setting of major trauma. Therefore, to account for the effect of stature on cross-sectional area, the ratio of the average P-CSA to the vertebral body CSA at the level of L4 was used. This is the PLVI, a measure of central sarcopenia that has been validated in previous studies [15, 16], including trauma populations [17]*.* Patients in whom bilateral measurements of P-CSA were not measurable were excluded. Standardised cut-offs for sarcopenia have not been validated; therefore, sarcopenia was defined as M-CSA and PLVI in the lowest quartile in respective patient subgroups.

### Outcomes

The primary endpoint was all-cause mortality within 2 years of initial presentation to the trauma centre. Patients who had no identifiable registered general practitioner, or who were lost to follow-up were excluded from analysis. Other co-variates recorded were age, injury severity score (ISS), need for intensive care admission, in-patient hospital complications (sepsis, acute kidney injury, myocardial infarction or decompensated heart failure, respiratory failure, venous thrombo-embolism), inpatient mortality, length of stay and discharge destination.

### Statistical analysis

Normally distributed data were compared using unpaired t test. Continuous skewed data were compared using the Mann–Whitney test. Values were expressed as the mean and standard deviations, or the median and interquartile ranges, respectively. Categorical comparisons utilised the Chi-squared test or in the case of discharge destination, the Fischer’s exact test. Binary outcomes were evaluated using logistic regression analysis and adjusted for age, ISS and gender. Log-scale analysis was used to ascertain differences in length of stay between sarcopenic and non-sarcopenic groups. Differences in outcomes were expressed as odds ratios. Cox regression for survival analysis and Kaplan–Meier curves were used to evaluate association of sarcopenia with survival times over 2 years. Statistical significance was set at *p* < 0.05.

## Results

### Sarcopenia based on M-CSA

One hundred and fifty five patients met the eligibility criteria including satisfactory visualisation of both masseters (Table 1). Thirty-nine patients (lowest quartile) had masseter sarcopenia, with M-CSA of 520 mm^2^ or lower. Nearly two-thirds of sarcopenic patients were female (73%, p < 0.001). There was no difference in age or injury severity score between the 2 groups (Table 2). Eight patients were excluded from 2-year follow-up due to missing data. Thus, 2-year mortality data were available for 147 patients (Table 1). There was no statistical association between masseter sarcopenia status and in-hospital complications or length of stay (Table 3). No significant differences were observed in 2-year survival or discharge destination between patients with and without masseter sarcopenia (Table 3).

**Table 1:** Exclusion characteristics for patients in masseter & PLVI groups

	Masseter	PLVI
Overall CT images (*n*)	257	241
*Exclusion*		
Images not visualized/artifact	82 (31.9%)	15 (6.2%)
Unilateral images only	6 (2.3%)	1 (0.4%)
No injury sustained	14 (5.4%)	20 (8.3%)
Statistical outlier	0	1 (0.4%)
Total number in data analysis	155 (60.3%)	204 (84.6%)
No registered GP in UK identified	8	6
Total number in 2-year follow-up	147	198

**Table 2:** Demographics and discharge destination of patients with and without masseter and PLVI sarcopenia

	Masseters			PLVI		
	Sarcopenia	No sarcopenia	*P*- value	Sarcopenia	No sarcopenia	*P*- value
*N*	39	116		55	149	
Age (*y*) (SD)	79.7 ± 7.4	77.0 ± 7.7	0.06	81.8 ± 8.9	76.7 ± 7.1	< 0.001
*Sex*						
Males	14 (36%)	85 (73%)	< 0.001	17 (31%)	105 (70%)	< 0.001
Females	25 (64%)	31 (27%)	–	38 (69%)	44 (30%)	–
ISS [IQR]	16 [5, 24]	16 [9, 26]	0.16	18 [9, 26]	16 [8, 25]	0.31
*Discharge destination*						
Home	21 (54%)	62 (53%)	1.00	16 (29%)	87 (58%)	**0.001**
Rehab Unit	3 (8%)	8 (7%)	–	4 (7%)	13 (9%)	–
Nursing Home	0 (0%)	1 (1%)		1 (2%)	3 (2%)	
Other hospital	7 (18%)	23 (20%)		16 (29%)	27 (18%)	
Died	8 (21%)	22 (19%)		18 (33%)	19 (13%)

**Table 3:** Comparison of outcomes between patients with and without masseter sarcopenia

Outcome	Analysis	Sarcopenia (*N* = 39) *n* (%)	No Sarcopenia (*N* = 116) *n* (%)	Odds Ratio ^#^(95% CI)	*P*-value
ICU admission	Unadjusted	3 (8%)	21 (18%)	0.38 (0.11, 1.34)	0.13
	Adjusted *	–	–	0.41 (0.09, 1.78)	0.23
Respiratory failure	Unadjusted	10 (26%)	35 (30%)	0.80 (0.35, 1.81)	0.59
	Adjusted *	–	–	1.03 (0.43, 2.53)	0.95
MI or heart failure	Unadjusted	1 (3%)	4 (3%)	0.74 (0.80, 6.80)	0.79
	Adjusted *	–	–	0.49 (0.04, 5.56)	0.57
Acute Kidney Injury	Unadjusted	33 (8%)	13 (11%)	0.66 (0.18, 2.45)	0.54
	Adjusted *	–	–	0.63 (0.15, 2.59)	0.52
Venous	Unadjusted	0 (0%)	3 (3%)	+	0.57
Thrombosis	Adjusted *	–	–	–	–
Sepsis	Unadjusted	4 (10%)	15 (13%)	0.77 (0.24, 2.47)	0.66
	Adjusted *	–	–	1.30 (0.36, 4.68)	0.69
Any complication	Unadjusted	15 (38%)	46 (40%)	0.95 (0.45, 2.00)	0.90
	Adjusted *	–	–	1.13 (0.50, 2.55)	0.77
In-hospital mortality	Unadjusted	8 (21%)	22 (19%)	1.10 (0.45, 2.73)	0.83
	Adjusted *	–	–	1.18 (0.56, 5.05)	0.35
Total length of stay (median days & IQR)	Unadjusted	13 [2, 22]	10 [4, 20]	0.94 (0.64, 1.36)	0.73
	Adjusted *	–	–	1.07 (0.73, 1.59)	0.72
		(N = 38)	(N = 109)	Hazard Ratio^#^	
2-year Survival time	Unadjusted	17	36	1.47 (0.82, 2.61)	0.19
	Adjusted ^(*)^	–	–	1.76 (0.94, 3.31)	0.08

*Adjusted for: age, sex, ISS

^#^Calculated as odds of outcome in Sarcopenia group relative to odds in No Sarcopenia group

^+^No occurrences in one category. Analysis using Fisher’s exact test

### Sarcopenia based on PLVI

**Fig. 1:**
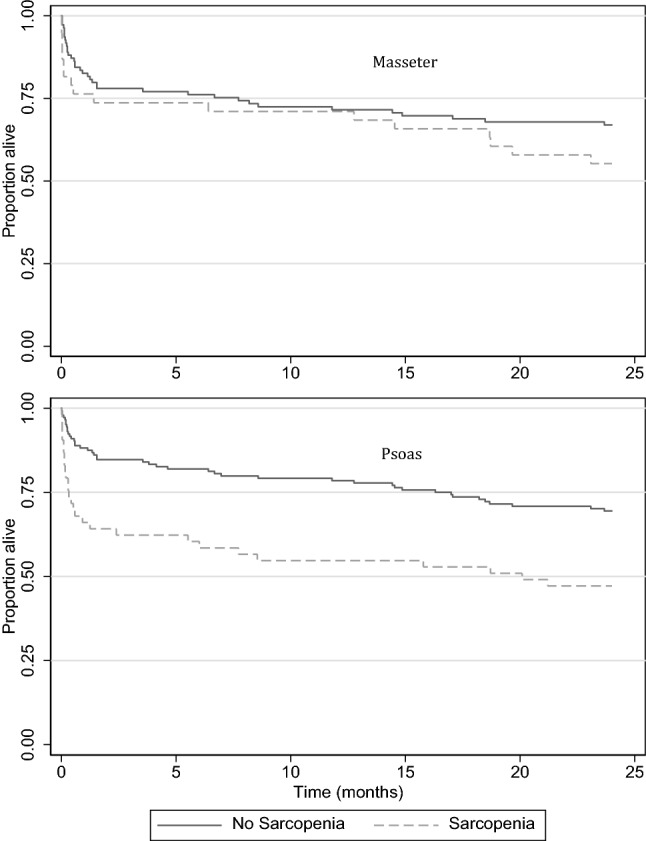
Kaplan–Meier plots of survival in patients with and without masseter (above) and psoas (below) sarcopenia

Two hundred and five patients were eligible for analysis (Table 1). One patient was excluded as an extreme outlier. Analysis was thus based on data from 204 patients, with a mean PLVI of 0.66 ± 0.19. Fifty-five patients (lowest quartile) who had PLVI values of 0.53 or lower were classified as sarcopenic. Two-year mortality data were available in 198 patients (Table 1). There was a statistically significant difference in gender between both groups, with 70% of females being sarcopenic (*p* < 0.001) (Table 2). Sarcopenic patients were older, with an average age of 82 years compared to 72 years in the non-sarcopenic group (*p* < 0.001) (Table 2). In-hospital mortality was significantly higher in sarcopenic patients (adjusted OR 3.38, 95% CI 1.47–9.73, *p* = 0.006) with 33% of patients with PLVI sarcopenia dying as inpatients compared to 13% of non-sarcopenic patients (Table 4). In-hospital complications were similar between groups. Only 29% of patients with PLVI sarcopenia were discharged home, compared to 58% in the non-sarcopenic group (*p* = 0.001) (Table 2). Patients with sarcopenia had shorter survival times, as illustrated by Kaplan–Meier survival curves (Fig. 1). The risk of death up to 2 years after injury in the sarcopenic group was 1.9 times higher after accounting for patient demographics and injury severity (adjusted OR 1.90, 95% CI 1.11–3.25, *p* = 0.02) (Table 4).

**Table 4:** Comparison of outcomes between patients with and without psoas sarcopenia

Outcome	Analysis	Sarcopenia (*N* = 55) n (%)	No Sarcopenia (*N* = 149) n (%)	Odds ratio ^#^(95% CI)	*P* value
ICU admission	Unadjusted	10 (18%)	21 (14%)	1.35 (0.59, 3.09)	0.47
	Adjusted *	–	–	1.37 (0.48, 3.93)	0.56
Respiratory failure	Unadjusted	16 (29%)	41 (28%)	1.09 (0.55, 2.14)	0.82
	Adjusted *	–	–	1.04 (0.47, 2.28)	0.93
MI or heart failure	Unadjusted	6 (11%)	4 (3%)	4.44 (1.20, 16.4)	0.03
	Adjusted *	–	–	2.52 (0.55, 11.5)	0.23
Acute Kidney Injury	Unadjusted	5 (9%)	17 (11%)	0.78 (0.27, 2.22)	0.64
	Adjusted *	–	–	0.50 (0.15, 1.71)	0.27
Venous Thrombosis	Unadjusted	1 (2%)	4 (3%)	0.67 (0.07, 6.14)	0.72
	Adjusted *	–	–	0.50 (0.04, 5.86)	0.58
Sepsis	Unadjusted	8 (15%)	24 (16%)	0.89 (0.37, 2.11)	0.79
	Adjusted *	–	–	0.63 (0.23, 1.73)	0.37
Any complication	Unadjusted	25 (45%)	58 (39%)	1.31 (0.70, 2.44)	0.40
	Adjusted *	–	–	1.08 (0.52, 2.21)	0.84
In-hospital mortality	Unadjusted	18 (33%)	19 (13%)	3.33 (1.59, 6.98)	0.001
	Adjusted *	–	–	3.38 (1.47, 9.73)	0.006
Total length of stay (days) [median, IQR]	Unadjusted	14 [6, 24]	10 [4, 18]	1.24 (0.90, 1.70)	0.19
	Adjusted *	–	–	1.21 (0.85, 1.71)	0.29
		(N = 53)	(N = 244)	Hazard Ratio ^(#)^	
2-year Survival time	Unadjusted	25	44	2.19 (1.36, 3.52)	0.001
	Adjusted *	–	–	1.90 (1.11, 3.25)	0.02

*Adjusted for: age, sex, ISS

^#^Calculated as odds of outcome in Sarcopenia group relative to odds in No Sarcopenia group

^+^Unable to calculate odds ratios, or perform logistic regression, due to no occurrences in one category. Analysis using Fisher’s exact test

### Association between M-CSA and PLVI

**Fig. 2:**
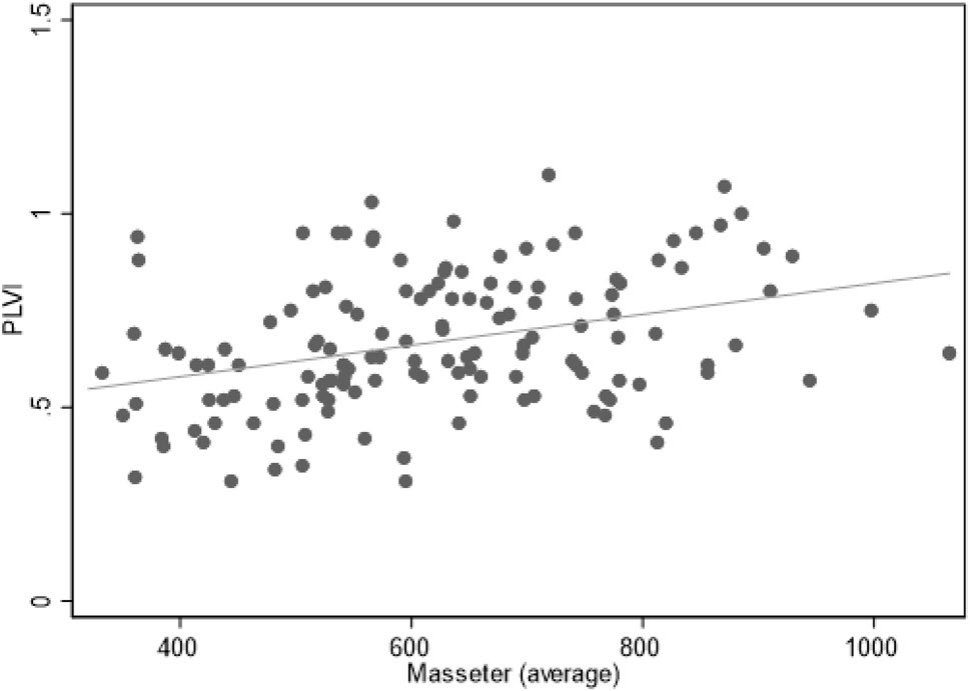
Scatterplot illustrating association of PLVI and Masseter measurements

One hundred and forty two patients had both M-CSA and PLVI measurements available. These had a positive linear relationship (Fig. 2), but the strength of association was weak (Pearson’s correlation coefficient 0.35, *p* < 0.01). While patients who had masseter sarcopenia were also more likely to have psoas sarcopenia (Table 5), discordancy was observed in 23% of the patients.

**Table 5:** Association between Psoas and Masseter sarcopenia definitions using Chi-squared test (*p* < 0.001)

Variable		Sarcopenia (psoas)		Total
		No	Yes	
Sarcopenia (masseter)				
No		90	16	106
Yes		17	19	36
Total		107	35	142

## Discussion

This is the first study in a UK elderly trauma population that examines both masseter and psoas muscle groups as indices for sarcopenia and their association with outcome. The findings of the study do not support our initial hypothesis; M-CSA is not proportionately comparable to P-CSA in predicting health outcomes in older trauma patients.

The odds of inpatient mortality are three times higher for older trauma patients with PLVI sarcopenia compared to those without. We also found that PLVI sarcopenia is an independent risk factor for reduced survival two years following injury and is associated with reduced likelihood of being discharged home. Our findings are consistent with several previous studies examining psoas sarcopenia in trauma populations [4, 7].

There was no statistical association between sarcopenia defined by either muscle group and inpatient complications or length of stay. Our study, like most others, uses muscle size as a proxy for sarcopenia. The operational definition of sarcopenia encompasses muscle quality and more importantly, physical strength. Overlooking these dimensions may explain why we did not note any prognostic associations with these important clinical outcomes [1].

Correlation between M-CSA and PLVI was weakly positive but M-CSA was not a predictor of overall mortality or any other measured health outcomes in our population. Our results contradict findings from other studies that have reported a positive association between M-CSA and mortality at different time points in the trauma population [11–13]. There may be several reasons for this. For example, in our study, the average CSA in patients with masseter sarcopenia was much higher— 438.5 ± 49.1 mm^2^ in females and 420.7 ± 70.4 mm^2^ in males. Two other studies quoted average sarcopenic values as 224 mm^2^ [12] and 343 mm^2^ [11] in females, and 281 mm^2^ [12] and 418 mm^2^ [11] in males. This heterogeneity highlights the importance of establishing standardised cut-offs, ideally referenced by healthy, non-hospitalised populations to prevent variations and over-diagnosis.

There were also differences in stratification of sarcopenia; we defined sarcopenia as the lowest quartiles in masseter and psoas populations regardless of sex. Sarcopenia was more prevalent amongst females in both groups including the PLVI group, in whom we adjusted for body stature. Other studies have defined sarcopenia with sex-based cut-offs below the median [13] or one standard deviation below mean [12].

Furthermore, our results may be impacted by exclusion bias; 34.2% of patients were excluded from statistical analysis due to inadequate visualisation of bilateral masseters, compared with only 6.6% in the PLVI group (Table 1). We may have failed to capture patients with reduced muscle quality. M-CSA was measured along the longitudinal axis, which requires reconstructing the imaging plane to align with the proximal and distal attachments. If accurate M-CSA measurement relies on higher-quality imaging or is technically more challenging, its viability as a metric for sarcopenia may be limited.

On post hoc analysis, our study is not sufficiently powered to show the observed 12% 2-year survival difference between the two masseter sub-groups. An estimated sample size of 605 patients would be required to reduce the probability of a type II error.

This study, nevertheless, suggests that M-CSA is not as robust a prognostic indicator as PLVI in these patients. Furthermore, localized masseter muscle atrophy can occur in patients with tooth loss, dental prosthesis or other causes for reduced masticatory function [9, 18, 19]. Caution needs to be exercised in using it as a surrogate for global muscle deconditioning. Masseter composition is also affected by body surface area [9] and craniofacial structure [20, 21]*.* Adjusting for body habitus and stature has relevance in achieving reliable sarcopenia measurements. Height and weight can be used to achieve this, but in the acute clinical setting, such as major trauma or emergency surgery, where the clinical application of sarcopenia measurement lies in augmenting emergent decision-making and prognostication, accurate height and weight measurements may not be readily available. Thus, sarcopenia measurement should ideally rely upon independent predictors of stature that can be measured on the same opportunistic imaging modality. We adjusted for this in our study using the L4 vertebral body CSA as part of the PLVI, but we are not aware of an available target for adjustment for stature in masseter sarcopenia quantification. Alternatively, combining CSA measurement with other metrics of masseter composition— be it, masseter volume, thickness or density— may increase sensitivity [22].

Our study is limited by virtue of its retrospective, single-centre design. We adjusted for injury severity, but confounders, such as comorbidity index, ethnicity and any operative interventions, were not examined. We did not measure the inter-rater reliability for the muscle CSA measurements. While psoas is the most commonly used muscle group in radiological evaluation of central sarcopenia in the trauma population [7], there is only one other study that looks specifically at PLVI [17]*.* Conversely, this showed an association of PLVI with morbidity but not in-hospital mortality. Differences in PLVI cut-offs and determinants of inpatient morbidity could explain this disparity.

The prospect of muscle segmentation on volumetric CT imaging using deep learning algorithms provides exciting opportunity for further work in this area and may overcome many of the challenges in sarcopenia measurement, improving precision and validity [23, 24]. The trauma population is unique in the challenges it imposes given the heterogeneity of injuries inflicted— in terms of severity, quantity and distribution of affected body areas. This makes prognostication and clinical decision-making more difficult. Given that many patients in a trauma or neurosurgical setting only undergo CT imaging of the head or neck, it is crucial that future studies focus upon cranial as well as abdominal imaging modalities to develop pragmatic clinical applications for opportunistic sarcopenia assessment. Some studies have indicated that morphometric analysis of temporal muscle thickness [25–27] or zygomatic thickness [25] may be suitable craniofacial surrogates of central sarcopenia. Composite analysis of all facial muscles may serve to enhance diagnostic accuracy. Combining sarcopenia as an objective metric with clinical frailty scoring may allow multi-dimensional frailty assessment that can augment prognostication and clinical decision-making. It may serve to identify patients that will benefit most from multi-disciplinary interventions and navigate decision-making around procedural interventions, discharge planning and palliation.

## Conclusion

Our study provides robust support that older patients with psoas sarcopenia are more likely to die in hospital and at 2 years and are less likely to be discharged to their home environment. As far as we are aware, this is the only study in the elderly general trauma population that did not find any prognostic relevance of M-CSA. This study poses important questions on the applicability of M-CSA to classify sarcopenia.

While our study has limitations, acknowledging these findings is important to direct future research. Incorporating multi-modal assessment of masseter muscles or inclusion of other craniofacial muscles for sarcopenia assessment may prove more reliable in enabling clinical application to elderly trauma populations.

## Data Availability

Datasets generated and analysed are available from corresponding author upon request.
